# Understanding the effects of decompaction maintenance on the infill state and play performance of third-generation artificial grass pitches

**DOI:** 10.1177/1754337114566480

**Published:** 2015-09

**Authors:** Paul R Fleming, Stephanie E Forrester, Nicholas J McLaren

**Affiliations:** 1 Civil and Building Engineering, Loughborough University, Loughborough, UK; 2 Wolfson School of Mechanical and Manufacturing Engineering, Loughborough University, UK; 3 Centre for Innovative and Collaborative Construction Engineering, Loughborough University, Loughborough, UK

**Keywords:** Artificial turf, third-generation, hardness, infill bulk density, maintenance

## Abstract

Third generation artificial grass pitches have been observed to get harder over time. The maintenance technique of rubber infill decompaction is intended to help slow, or reverse, this process. At present, little is understood about either the science of the infill compaction process or the efficacy of decompaction maintenance. The objective of this study was to measure the changes in rubber infill net bulk density, force reduction (impact absorption) and vertical ball rebound under various levels of compactive effort in controlled laboratory-based testing. The assessments were repeated after the systems had been raked to simulate the decompaction maintenance techniques. These tests defined the limits of compaction (loose to maximally compacted) in terms of the change in rubber infill net bulk density, force reduction and vertical ball rebound. Site testing was also undertaken at four third generation pitches immediately pre and post decompaction, to determine the measurable effects in the less well controlled field environment. Rubber infill net bulk density was found to increase as compactive effort increased, resulting in increased hardness. Decompacting the surface was found to approximately fully reverse these effects. In comparison, the site measurements demonstrated similar but notably smaller magnitudes of change following the decompaction process suggesting that the field state pre and post decompaction did not reach the extremes obtained in the laboratory. The findings suggest that rubber infill net bulk density is an important parameter influencing the hardness of artificial grass and that decompactions can be an effective method to reverse compaction related hardness changes.

## Introduction

**Figure 1.**
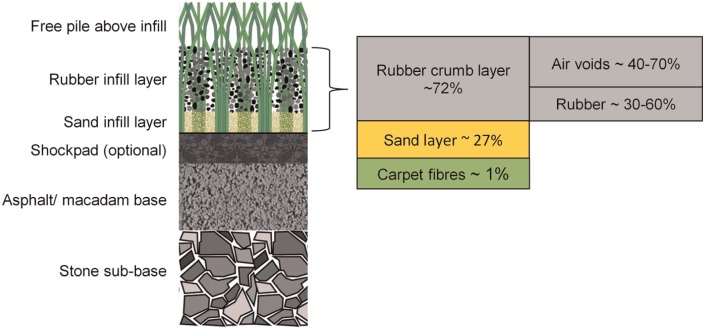
Cross section through a typical third-generation artificial grass pitch system. Schematic showing the relative volumes of the rubber crumb layer, sand layer and carpet fibres within carpet system. The rubber crumb layer can be further broken down into the relative volume of air and rubber, the ratio of which will depend on the state of infill compaction and determine the rubber infill net bulk density.

Third-generation artificial grass pitches (3G AGPs) are becoming increasingly common at community and competition levels for sports such as association football and rugby football. The carpet is made from polyethylene or polypropylene fibres, typically 35–70 mm in length, while particulate infill materials include a lower sand layer and an upper rubber crumb layer. A shockpad can be included beneath the carpet and the complete system is installed on a stable base of aggregate and/or a bound upper layer such as asphalt (Figure 1). For a new installation, or a controlled laboratory sample (free from contamination or the effects of use), the mass and particulate size range of the rubber and sand infills,^1,2^ their mass ratio,^1,3,4^ and the type and thickness of the shockpad^3,5^ have been shown to control the system hardness. Current Fédération Internationale de Football Association (FIFA)^6^ requirements include three measurements related to pitch hardness (Table 1). Force reduction (FR), measured using the Advanced Artificial Athlete (AAA), is the peak impact force from a controlled energy falling mass impact test and is expressed as a percent reduction relative to a rigid concrete surface. Vertical ball rebound (VBR) is expressed as the ball rebound height from a drop height of 2 m.

**Table 1.** Key hardness performance requirements from the FIFA^6^ Quality Concept for AGPs.

Test	Requirement		Test method
	FIFA 1*	FIFA 2*	
Force reduction (%)	55–70	60–70	FIFA 04a
Vertical deformation (mm)	4–11	4–10	FIFA 05a
Vertical ball rebound (m)	0.60–1.00	0.60–0.85	FIFA 01

The FIFA 1* category is designated for training and community use with an increased range for the testing requirements. The FIFA 2* category is designated for professional use with the upper and lower limits for each test defined to replicate good quality natural grass.

The mechanisms of AGP degradation are not well researched and the scale of effects not well understood due to the many variables and their complex interaction. The commonly observed degradation processes include fibrillation, fracture and flattening of the fibres (loss of resilience or support from the infill), compaction, migration or loss of the rubber infill and contamination (e.g. foreign material such as litter, leaves, soil, broken fibres) clogging up the infill layer or carpet drainage holes.^7,8^ There is strong evidence that these degradation processes cause AGPs to become harder over time/use (Table 2).^9–12^ Of these studies, the most comprehensive assessed 50 3G AGPs used for association football over a 7-year period in The Netherlands and found that on average FR decreased by 10% and VBR increased by 0.15 m over this time period, with little change in infill depth (−2 mm)^9^ There were also general trends for changes in hardness wherein pitches with increased usage and/or decreased maintenance demonstrated a greater decline in performance. In another study, 20 randomly selected AGPs, which specifically had not previously been certified by field testing and were of various ages, were evaluated in one season in Spain. The study found significant negative differences in performance between AGPs in three separate categories: age, that is, those <5 years old versus 5–10 years old; usage, that is, <35 versus >35 h per week and maintenance, that is, specific regular maintenance versus no maintenance.^12^ The study was limited by unknown variations in system design but provided further evidence, as might be expected, that age, intensity of use and maintenance all appear to have a real effect on play performance.

**Table 2.** Force reduction and vertical ball rebound from previous research tracking third-generation AGP hardness as a function of age.

Author	No. of AGPs	Test period (years)	Force reduction (%)			Ball rebound (m)		
			Start	End	Change	Start	End	Change
Jan-Kieft^9^	50	7	53.0	43.0	−10.0	0.82	0.97	+0.15
Joosten^10^	6	1	56.0	51.1	−4.9	0.88	0.83	−0.05
Sanchez-Sanchez et al.^11^	4	1	61.2	59.7	−1.5	0.85	0.88	+0.03
Burillo et al.^12, **a**^	10 per group	<5 years old versus 5–10years old	53.6	35.7	−17.9	0.81	1.14	+0.33

AGP: artificial grass pitch; FR: Force reduction; VBR: vertical ball rebound.

The first three collected data from the same pitches at the start and end of the testing period. The final study measured hardness for two sets of pitch of contrasting age.

a In the Burillo et al.^12^ study, the data were gathered in one season so the data represent the average FR and VBR for the pitches in each age category and not a direct change over time.

Since AGPs represent a significant capital investment, it is relevant to maximise their service life which includes minimising the degradation in play performance properties over this period as well as ensuring a safe playing surface for the users. The factors that influence this include the original system design, quality of installation, intensity of use, local environmental factors and the maintenance regime applied to the pitch.^13–15^ Regular maintenance is increasingly seen as an important part in helping to maintain the play performance of a pitch over time through slowing down and/or reversing certain degradation processes. However, the effectiveness of the various maintenance processes is largely based on observation and experience, with little published data to corroborate the varying views and opinions.^14,16,17^

Decompaction maintenance processes are specifically aimed at reducing pitch ‘hardening’ caused by rubber infill compaction that occurs through mechanical working of the surface by the player interactions. If the surface becomes too hard, it may fail the field accreditation tests and require some investment and repair to bring it back to satisfactory standard. The aim of the decompaction process is to agitate the rubber infill layer causing it to decompact to a looser state. In brief, large rakes consisting of rows of metal tines are dragged across the surface behind a vehicle where the tine depth is set to penetrate the rubber infill layer. Typically, the rakes are dragged across the pitch width (to avoid crossing longitudinal seams) on a single or double pass and this process is completed every 1–2 months. This decompaction process can be more efficiently undertaken in conjunction with cleaning and grooming processes to limit downtime of the field. How to measure the effectiveness of decompaction is currently unknown, however, making it difficult to optimise the equipment design and/or how often it is carried out, or to determine site-specific decompaction needs.

To date, only limited research has been reported investigating the effects of infill compaction on infill state/changes in state (e.g. density) or play performance measurements. It has been shown that rubber crumb on its own (in a steel mould) compacts under repeated loading^3^ with the bulk density ranging from 0.48 g cm^−3^ (loosest state) to 0.59 g cm^−3^ (densest state). A higher density state was actually achieved under high-load static compression; however, this was mostly elastic recoverable strain (for the initially dense sample) and was recovered after unloading. Across a range of complete AGP systems, from the loosely installed state through to compaction of up to 200 cycles with a standard weighted studded roller,^18^ a wider range of rubber infill net bulk densities resulted (from 0.33 to 0.80 g cm^−3^). The absolute change in rubber infill net bulk density for a single system from the loosest to the densest state varied but was generally slightly larger than achieved in the steel moulds (by between 0.10 and 0.21 g cm^−3^). In this case, the rubber infill state was expressed by the rubber infill net bulk density, that is, the known mass per unit area of rubber crumb divided by the measured rubber crumb depth, with the volume contribution of fibres within this rubber infill layer removed.^3^ The same study also reported only a small decrease in FR, in the range of 0.1%–3.4% absolute, between the loosely installed infill state and densest infill state.^3^ Furthermore, this decrease in FR tended to be of lesser magnitude as shockpad thickness increased. A similar decrease in FR has been reported following compaction of 100 cycles of a standard roller.^2^ Compaction generated by the Lisport accelerated mechanical wear machine^18^ resulted in a similar decrease in FR and increase in VBR for seven 3G AGP systems.^1^ FR decreased by 2.3% absolute and VBR increased by 0.09 m following 200 cycles of compaction and by 4.5% and 0.15 m following 2000 cycles of compaction. These results suggest non-linearity in the effects of compaction on FR and VBR, with VBR in particular demonstrating a greater change over the initial 200 cycles compared to the remaining 1800 cycles. Unfortunately, infill depth was not reported for this testing and therefore, the specific changes in infill state cannot be estimated.

To date, there has been no field-based study investigating the effects of infill compaction on play performance. The challenges of such a study include the difficulty in quantifying infill state (rubber infill net bulk density) since the mass per unit area of rubber crumb is unknown and only the total infill depth can be measured (i.e. sand layer and rubber layer combined). Even where the manufacturer’s specifications for the field are available, this typically only provides guidelines for the quantities of infill added (kg m^−2^) and does not account for subsequent migration of infill across the field that occurs with use. Furthermore, numerous variables, in addition to infill state, are thought to affect the hardness response including moisture level, temperature, contamination levels and shockpad thickness/density. However, it may be considered that testing hardness pre and post decompaction maintenance has the potential to yield useful results, particularly if the change in infill state, that is, depth, is sufficiently large and/or hardness measurement techniques utilised are sufficiently sensitive to these changes.

The purpose of this study was to investigate the compaction/decompaction hardness behaviour of 3G artificial grass under both controlled laboratory and real conditions. The specific objectives were as follows:

1. To investigate the effects of different levels of compaction on the rubber infill state (net bulk density) and play performance (FR and VBR) for a 3G AGP system under controlled laboratory test conditions.
2. To investigate the effect of decompaction maintenance on play performance properties of real AGPs and how this can be best measured.
3. To compare the laboratory and field data in order to improve our understanding of how infill state and play performance can be assessed on site to support the scientific development of pitch monitoring and maintenance with respect to hardness.

## Methods

Laboratory testing was conducted to investigate controlled density changes in the rubber infill in order to assess the range of density change that can be achieved and to elicit the impact of this density change on measurements of FR and VBR. Field testing was completed at four selected sites to determine the effects of the full-scale decompaction process on total infill depth, FR and VBR. This provided a consistent sample of fields that generated data that could be discussed in the context of the laboratory findings.

### Laboratory testing

#### Carpet preparation

A 65-mm monofilament carpet with sand and the rubber infill was selected for the laboratory testing (Table 3). The manufacturer’s recommended spread rate of 13 kg m^−2^ of 2EW sand (size range: 0.2–0.7 mm) and 14 kg m^−2^ of styrene butadiene rubber (SBR) rubber (size range: 0.5–2.5 mm) were applied, pro rata, to the 0.5 × 0.75 m carpet sample. Based on previous experience of generating surface samples in the laboratory, the infill materials were applied to the surface in 2 kg batches while regularly measuring the infill depth to ensure even coverage. Once the sand layer had been applied, it was conditioned using 50 cycles of a weighted studded roller after which the infill depth was measured in 24 positions across each sample giving agreement to within ±1 mm. After each batch of rubber was applied, the surface was raked to distribute the infill evenly across the surface.

**Table 3.** Summary of the surface systems used in the laboratory and field.

Component	Properties	Laboratory	Field sites			
			W	CH	CR	B
Carpet fibres	Length (mm)	65	60	60	60	60
	Mono/fibrillated	Mono	Mono	Mono	Mono	Mono
Sand infill	Mass (kg m^−2^)	12.8	13^a^	14^a^	–	22.5^a^
	Depth (mm)	15				
	Particle size (mm)	0.2–0.7	0.3–0.8	0.4–0.8	–	0.5–1.0
Rubber infill	Mass (kg m^−2^)	13.9	17^a^	15^a^	–	16^a^
	Depth (mm)	31				
	Particle size (mm)	0.8–2.5	0.5–2.5	0.8–2.5	–	0.8–2.5
Site specific	Built (year)		2011	2013	2011	2010
	Substrate	Concrete	Tarmacadam/asphalt	Tarmacadam/asphalt	Tarmacadam/asphalt	Stone
	Shockpad		No	No	No	No
	Usage (h/week)		33	45	40	35–40
Maintenance schedule	Drag brushing		Weekly in-house	Weekly in-house	Weekly in-house	Weekly in-house
	Sweeps		6 per year	6 per year	6 per year	6 per year
	Decompactions		3 per year	3 per year	3 per year	3 per year

a For the field sites, the sand and rubber mass details were taken from the manufacturer’s specification.

#### Compaction

A standard weighted studded roller was used to compact the rubber infill. The roller design matched that used in the commercial laboratory accreditation of artificial grass systems, being 400 mm wide and having a total mass of 43.6 kg.^3,18^ Three levels of infill compaction were investigated (0, 50 and 500 cycles), based on previous testing observations that showed only a limited decrease in FR occurred following 200 cycles with the same roller.^3^ It was expected that 500 cycles would ensure that the maximum achievable increases in infill net bulk density and decreases in FR had occurred. One cycle equated to two passes of the roller across the surface, one outward and one return.

#### Decompaction

A regular garden leaf rake was used to thoroughly decompact the rubber infill back to a loose state (it was not aimed at replicating site decompaction energy or force). The rake comprised a row of 20 metal tines 195–215 mm long, 3.2 mm in diameter, spaced 23 mm apart (total width: 450 mm). The rake was pulled through the surface, with the force manually applied by the same operator in a consistent manner throughout the trials, in both vertical and horizontal directions to ensure the tines were penetrating into the rubber infill by approximately 20 mm. The surface was raked lengthwise from both ends of the sample and side-to-side from both sides of the sample to ensure even distribution of the rubber infill following decompaction, with two passes in each direction. This method was chosen after trialling a range of methods for returning infill depth to its original (pre-compaction) levels following compaction.

#### Measurements

**Figure 2.**
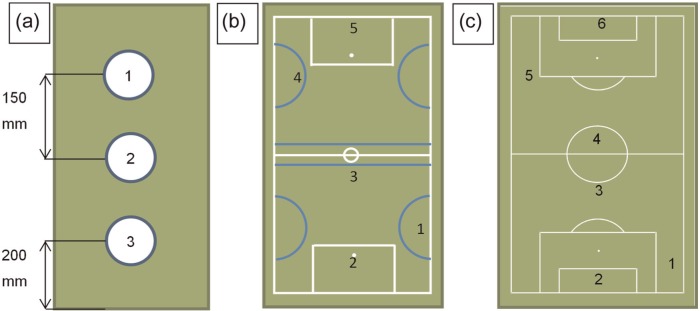
Testing locations for (a) the laboratory testing, (b) sites W and B and (c) sites CH and CR.

Measurements of total infill depth, FR and VBR were taken at three locations on each sample (Figure 2(a)). Total infill depth was measured using Vernier calipers and was used to estimate the rubber infill net bulk density according to^3^

(1) $$\rho =\frac{m}{V_{\mathrm{sys}}-V_{f}}$$

where *ρ* is the rubber infill net bulk density, *m* is the mass of the rubber infill, *V_sys_* is the volume of the surface system (area × height of the rubber infill) and *V_f_* is the volume of fibres (number fibres × width of a fibre × thickness of a fibre × height of a fibre interacting with the measured infill depth). This calculation assumed that the depth of the sand layer remained constant, and that the system was dry and free from contamination.

FR was measured using the AAA.^18^ The AAA is a standardised apparatus to determine the FR of a sports surface. A 20 ± 0.05 kg mass is dropped from a height of 55 ± 0.25 mm onto the surface. The mass is attached to a spring and test foot to form a damped impact while an accelerometer mounted on the mass allows the impact characteristics to be quantified. Each test consisted of 10 impacts of the AAA allowing the full hardening profile of the surface to be captured. FR typically reduces with increasing impacts in an exponential manner with 10 impacts required to reach the ‘hard’ plateau. Since infill state is the focus of this study, and the characteristics of this hardness decay are likely to be influenced by the infill state it was considered important to capture this full decay. Infill depth measurements were taken on the impact location prior to the 1st impact and after the 10th impact only, to avoid disturbance to the infill during the intermediate impacts.

One VBR measurement^18^ was taken at each of the three locations across the sample, with three infill depth measurements made in the approximate impact position prior to the ball rebound (Figure 2(a)). A FIFA-approved football was used, whereby the pressure was adjusted so that the ball rebounded 1.35 ± 0.03 m on the laboratory concrete floor from a drop height of 2.0 m. Rebound height was determined acoustically from the timeframe between the first and second bounces using the following equation^18^

(2) $$H=1.23{\left(T-\Delta t\right)}^{2}\times 100$$

where *H* is the VBR height (m), *t* is the time between the first and second bounce (s) and Δ*t* is the correction factor for footballs (0.025 s).

#### Protocol

Three identical carpet samples were created; one for each level of compaction. For the AAA testing, infill depth measurements were taken on the starting sample with the infill in a loose state. The infill was then compacted using the set number of cycles with the studded roller. Following this process, infill depth measurements were taken, 10 impacts of the AAA conducted and infill depth measurements repeated. The infill was then decompacted using the raking procedure. Following this process, infill depth measurements were taken, 10 impacts of the AAA conducted and infill depth measurements repeated. Following raking, a similar procedure was repeated for VBR. In all cases, the infill depth measurements were taken at the expected impact location of the AAA/ball.

### Field testing

#### Test sites

Four sites of similar system design were selected to investigate the effects of the decompaction maintenance process (Table 3). Even though the systems were considered to be of standard design, they were supplied by different carpet manufacturers and therefore may have subtle differences. All were relatively new (1–3 years old) and had undergone regular maintenance since first installed. During testing, the weather conditions at each site were similar; the surface was damp with a temperatures ranging between 5.2 °C and 11.8 °C.

#### Measurements

Five/six test locations were selected at each site (Figure 2(b) and (c)) including expected high- and low-use areas of the pitch.^6,19^ FR measurements were taken using a club tester (CT; Deltec Equipment, RZ Duiven, The Netherlands). This portable tool is used for determining the FR of a surface giving directly comparable values to those from the AAA and permitting much quicker site data collection. Three FR measurements were carried out at each test location with each measurement involving 10 impacts of the CT as for the laboratory testing with the AAA. Five VBR measurements were carried out at each test location using the same protocol to the laboratory testing. Total infill depth was measured in the CT impact position and prior to each VBR measurement again at the expected impact location. These measurements were conducted in the hour before the decompaction maintenance was carried out and repeated in the hour after to assess the effect of the decompaction on the surface state and hardness.

#### Decompaction maintenance

The decompaction maintenance treatment varied slightly between fields, as the testing occurred alongside contracted works. Site W had the most invasive procedure with six passes (covering a range of directions) of the maintenance machine. The decompaction rake was 1.1 m wide and had 6 rows of 22 tines arranged in an offset pattern. The individual tines were 10 mm in length and 2.7 mm in diameter and angled at approximately 10° to the vertical. The depth of penetration was progressively increased during the maintenance procedure by the operators, as they had been tasked with fully decompacting the surface at site W. Sites CH, CR and B received only a single pass of the maintenance machine. The maintenance machine used at site CR and B was the same as at site W (SportsChamp SC3D, SMG, Vöhringen, Germany); however, site CH used a slightly different machine (CareMax CM2D, SMG, Vöhringen, Germany). In this case, the tines were arranged in only two rows; however, the tine dimensions and distance between tines remained the same.

#### Statistical analysis

To test for significant differences in total infill depth, FR and VBR between the compacted and decompacted conditions (pre and post decompaction maintenance) Wilcoxon signed rank tests (SPSS Inc., Chicago, IL, USA) were undertaken. This non-parametric test was used as the laboratory data contained small sample sizes and the field data were not normally distributed (based on Shapiro–Wilk testing). To investigate the relationship between rubber infill state and hardness of the surface, Pearson’s correlation coefficient was determined for the rubber infill net bulk density (laboratory)/total infill depth (field) and both FR and VBR. The relationship was assumed to be strong for |r| ≥ 0.5, moderate for 0.5 > |r| ≥ 0.3 and weak for |r| < 0.30.^20^ In all cases, significance was set at *p* ≤ 0.05.

## Results

### Laboratory testing

**Figure 3.**
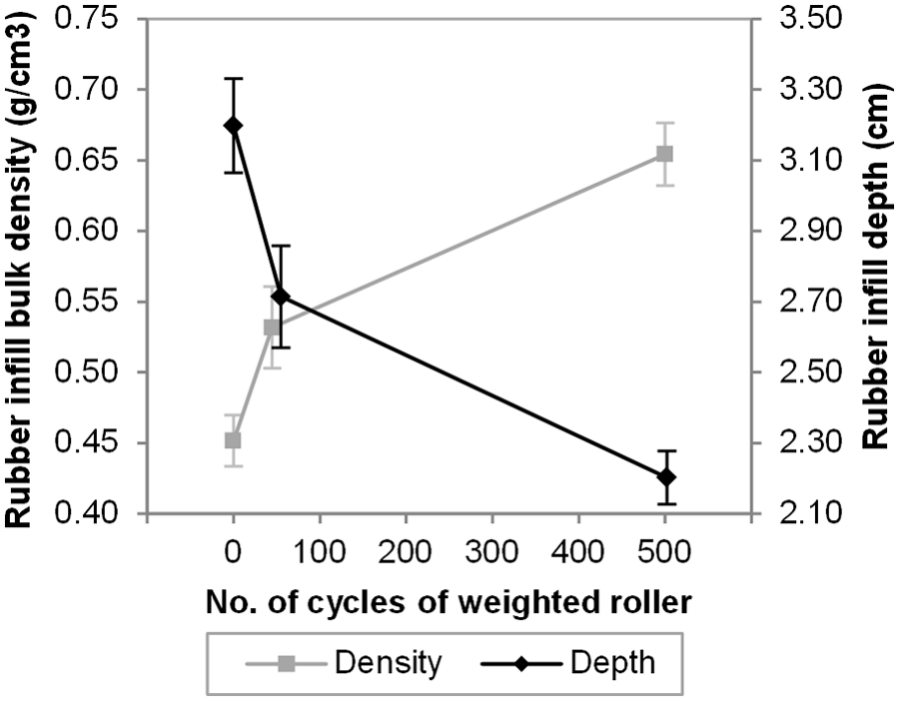
Mean ± 1 standard deviation in rubber infill depth and rubber infill net bulk density as a function of number of cycles with the weighted studded roller.

**Figure 4.**
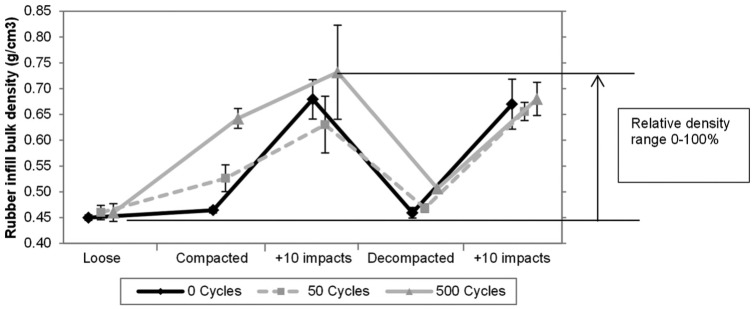
Rubber infill net bulk density at each stage of testing for the three different levels of compactive effort: (1) loose state, (2) following roller compaction, (3) following roller compaction and 10 impacts of the AAA, (4) following decompaction (raking) and (5) following decompaction (raking) and 10 impacts of the AAA.

**Figure 5.**
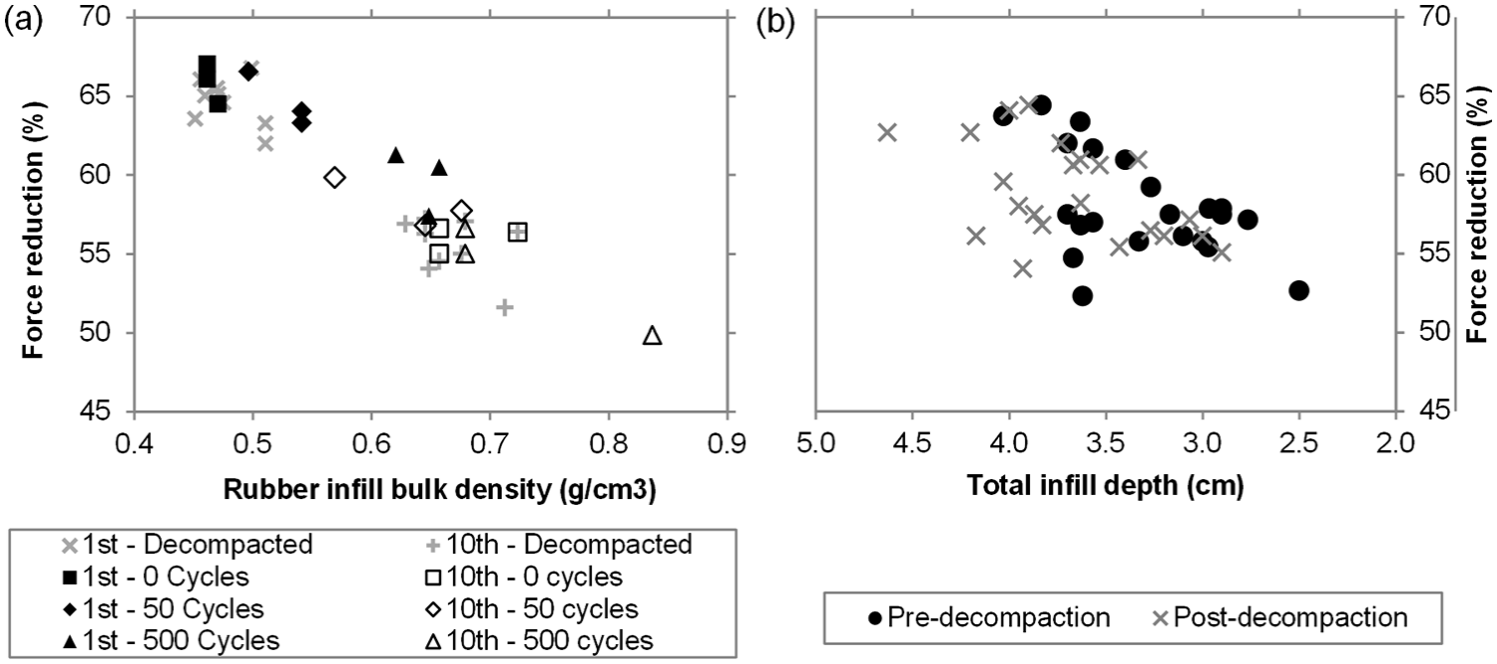
(a) Force reduction versus rubber infill net bulk density from laboratory testing. The data are from pre-1st impact and post-10th impact of the AAA immediately following the three levels of compactive effort and pre-1st impact and post-10th impact of the AAA immediately following the decompaction raking. (b) Force reduction versus total infill depth from the four field sites. The data are from pre-1st impact of the CT before (compacted, black) and after (decompacted, grey) the decompaction maintenance.

**Figure 6.**
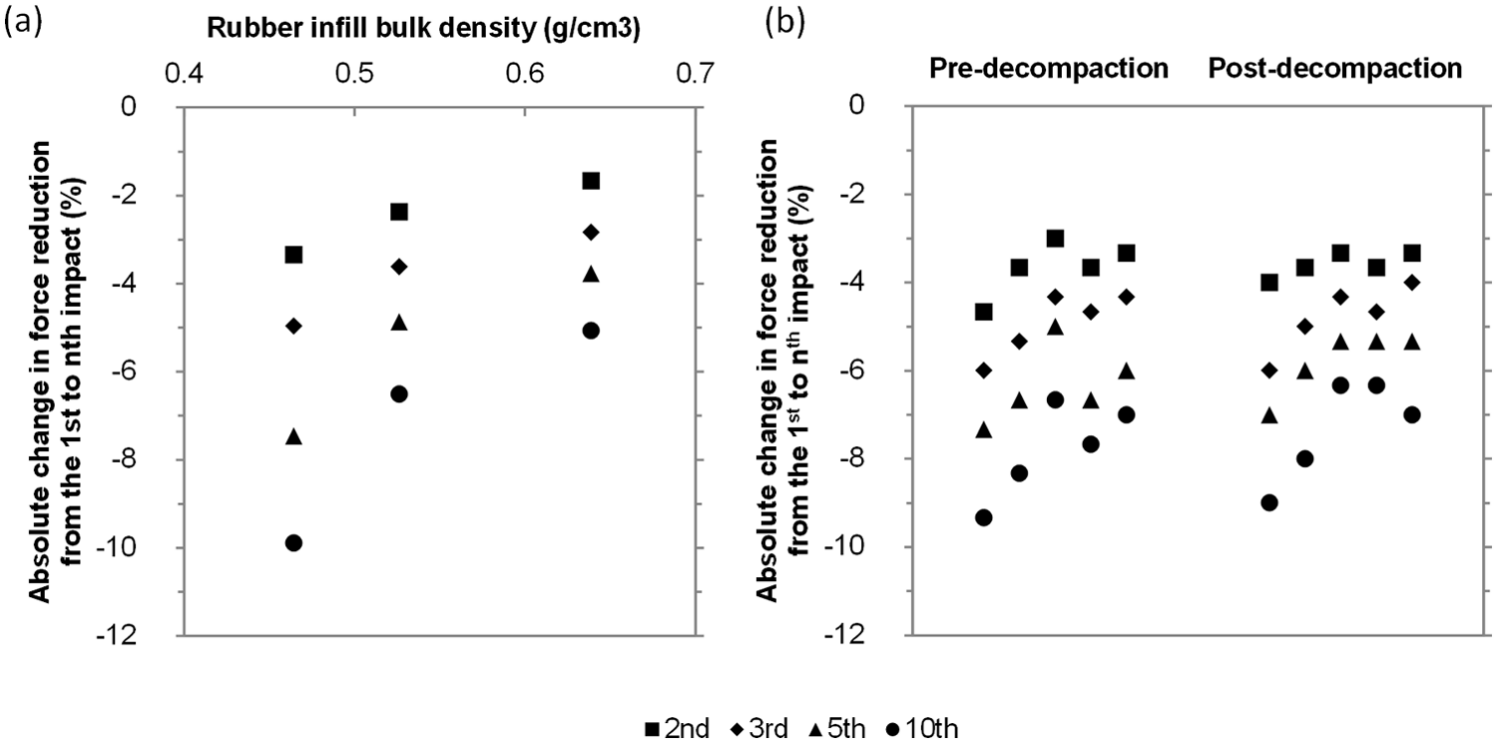
(a) Absolute change in force reduction from the 1st to nth impact (n = 2, 3, 5, 10) for the three different compactive efforts (0, 50 and 500 cycles) from the laboratory testing. (b) Absolute change in force reduction from the 1st to nth impact (n = 2, 3, 5, 10) for the pre- and post-decompaction maintenance from the five test locations at field site CH.

**Figure 7.**
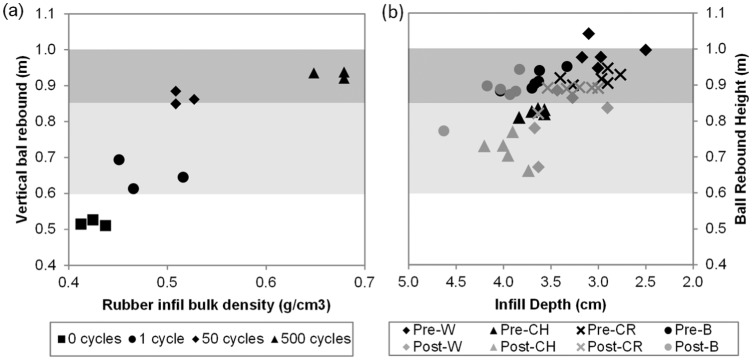
(a) Vertical ball rebound versus rubber infill net bulk density from the laboratory testing for the different compactive efforts. (b) Vertical ball rebound versus total infill depth from the four field sites. The data are from before (compacted, black) and after (decompacted, grey) the decompaction maintenance. The complete shaded area represents the FIFA 1* limits while the lighter shaded area represents the FIFA 2* limits.

The results from the laboratory testing are presented in Table 4 and Figures 3, 4, 5(a), 6(a) and 7(a). In brief, these confirm that the testing methods achieved a wide range of infill states from the raked loose state to the rolled and AAA highly compacted state, and that this resulted in highly measurable differences in surface hardness quantified by both FR and VBR.

**Table 4.** Average force reduction, ball rebound and infill depth data at different stages of the laboratory and site testing.

		Laboratory			Field sites			
		0 cycles	50 cycles	500 cycles	W	CH	CR	B
Force reduction (%)								
Compacted	1st impact	65.9 ± 1.3	64.6 ± 1.7	59.7 ± 2.1	55.5 ± 1.8	61.7 ± 2.8	58.4 ± 1.5	56.8 ± 3.8
	2nd/3rd impact	61.7 ± 1.4	61.6 ± 1.7	56.8 ± 3.3	51.0 ± 1.7	57.3 ± 3.4	54.9 ± 1.7	52.7 ± 3.6
Decompacted	1st impact	64.9 ± 1.3	65.1 ± 0.5	64.0 ± 2.5	57.2 ± 2.3	62.2 ± 2.6	58.7 ± 2.5	57.8 ± 3.0
	2nd/3rd impact	61.7 ± 0.6	61.2 ± 0.9	59.9 ± 2.8	52.4 ± 1.7	57.9 ± 3.1	55.4 ± 2.5	53.4 ± 3.2
Absolute change	1st impact	−1.0 ± 1.6	+0.4 ± 1.4	+4.3 ± 1.4	+1.7 ± 1.6	+0.5 ± 0.6	+0.5 ± 2.5	+0.8 ± 1.6
	2nd/3rd impact	**−0.1 ± 1.9**	**−0.4 ± 1.3**	**+3.1 ± 1.6**	**+1.3 ± 1.2**	**+0.6 ± 0.6**	**+0.7 ± 2.6**	**+0.6 ± 1.7**
Sig. (*p* = <0.05)	1st impact	**0.285**	**0.593**	**0.109**	**0.010**	**0.208**	**0.551**	**0.048**
	2nd/3rd impact	**1.000**	**0.593**	**0.109**	**0.010**	**0.179**	**0.337**	**0.147**
Vertical ball rebound (m)								
Compacted		0.52 ± 0.01	0.87 ± 0.02	0.93 ± 0.01	0.99 ± 0.03	0.82 ± 0.01	0.92 ± 0.02	0.91 ± 0.03
Decompacted		–	–	–	0.81 ± 0.08	0.72 ± 0.04	0.88 ± 0.03	0.88 ± 0.06
Absolute change		–	–	–	−0.18 ± 0.09	−0.11 ± 0.04	−0.04 ± 0.03	−0.04 ± 0.04
Sig. (*p* = <0.05)					<0.000	<0.000	0.004	<0.000
Infill depth (mm)		Rubber infill depth (mm)				Total infill depth (mm)		
Compacted		31.0 ± 0.3	27.4 ± 1.4	22.4 ± 0.7	29.5 ± 2.6	36.6 ± 1.1	30.4 ± 2.4	36.6 ± 2.2
Decompacted		31.3 ± 0.7	30.7 ± 0.5	28.4 ± 0.4	33.8 ± 3.2	39.6 ± 1.7	32.9 ±2 .5	40.8 ± 3.0
Absolute change		**+0.3 ± 0.3**	**+3.3 ± 1.5**	**+6.0 ± 0.3**	**+4.3 ± 1.8**	**+3.0 ± 2.3**	**+2.6 ± 2.3**	**+4.1 ± 1.6**
Sig. (*p* = <0.05)		**0.180**	**0.109**	**0.109**	**0.001**	**0.004**	**0.002**	**<0.000**
Rubber infill net bulk density (g** **cm^−3^)								
Compacted		0.46 ± 0.01	0.53 ± 0.03	0.64 ± 0.02				
Decompacted		0.46 ± 0.01	0.47 ± 0.01	0.51 ± 0.01				
Absolute change		**0.00 ± 0.01**	**−0.06 ± 0.03**	**−0.14 ± 0.01**				
Sig. (*p* = <0.05)		**0.317**	**0.109**	**0.109**				

Wilcoxon signed rank testing undertaken for statistical significance. Infill depth in the laboratory was the height of the rubber only, while on site the measurement refers to total infill depth (rubber and sand).

#### Rubber infill net bulk density

Rubber infill net bulk density increased and rubber infill depth decreased as the number of cycles with the weighted studded roller (compactive effort) increased (Figure 3). These relationships were non-linear with the majority of change occurring over the lower number of cycles, that is, almost half (44%) of the total increase in infill net bulk density was achieved in the first 50 cycles and similarly for infill depth.

In its original (loosest) state, the rubber infill net bulk density was approximately 0.45 g cm^−3^ which increased with compactive effort using the studded roller, and further still following the 10 impacts with the AAA, to its densest state of approximately 0.73 g cm^−3^ (Figure 4). This range of achievable rubber infill net bulk density can be termed the working, or relative, density range for the current system design and changes in bulk density referred to on this basis. Decompaction, through raking, returned the rubber infill net bulk density to 0%–15% of this relative density range (i.e. close to its original loose state), while even the 10 impacts with the AAA on the loose infill caused the density to increase to 85% of the relative density range, indicating the substantive compactive effort achieved by the AAA impacts.

#### FR

As the initial rubber infill net bulk density increased, that is, the rubber infill became more compacted, FR reduced from a maximum of 67% down to 50% (Figure 5(a)). The relationship was negative, linear, significant and strong (r = 0.93, *p* < 0.000), thus supporting the earlier observations, related to changes in rubber infill net bulk density with compactive effort, similarly hold for FR. Notably, this range of FR spans from near the top (soft) end of the FIFA 1* and 2* regulations to substantially beneath the bottom (hard) end (Table 1).

The absolute change in FR from the 1st to the nth impact (n = 2 → 10) of the AAA can provide further information related to the state of the infill given that FR tends to decrease exponentially with the number of drops. A similar trend was established with previous research utilising the head impact criterion hardness testing on 3G surfaces.^21^ By using 10 impacts, this allowed the entire decay curve to be quantified. The laboratory data illustrated that as the initial rubber infill net bulk density increased, the total range in the decay of FR (from the 1st to the 10th impact) decreased (see Figure 6(a) which, for clarity, shows selected drops only). When the AAA testing was completed on the carpet with the infill in its loosest state, FR changed by 10% absolute between the 1st and 10th impact, whereas when the AAA testing was completed on the carpet with the infill in close to its densest state, FR changed by only 5% absolute between the 1st and 10th impact. This illustrates that the effect of repeated impacts with the AAA is affected by the initial rubber infill net bulk density state and such testing may represent an alternative and improved method for differentiating infill state compared to the standard method of using the absolute FRs from only three impacts.

#### VBR

As the initial rubber infill net bulk density increased, that is, the rubber infill became more compacted, the VBR increased from a minimum of 0.51 up to 0.94 m (Figure 7(a)). The rate of increase was greatest at the lower densities and approximately linear; 0.38 m (from 0.51 up to 0.89 m) of the increase occurred over the first 33% of the relative density range and only 0.05 m over the remaining 67% of the relative density range. Hence, VBR appears sensitive to infill state and is thus potentially a good method for quantifying infill state, when the infill is relatively decompacted. At higher levels of infill compaction/bulk density, VBR appears less useful as it is relatively insensitive to infill state. This range of VBR spans from near or above the top (hard) end of the FIFA 1* and 2* regulations to substantially beneath the bottom (soft) end (Table 1).

### Field testing

The results from the field testing are given in Table 4 and Figures 5(b), 6(b) and 7(b). In brief, these provided evidence that the decompaction maintenance was successful in changing the infill state (increasing total infill depth by between 3 and 4 mm); however, the corresponding change in FR was small (<1%–2% absolute) while the change in VBR was small to moderate (0.04–0.18 m). Notably, the site (W) that underwent the more intensive decompaction displayed the largest changes in total infill depth, FR and VBR.

#### Total infill depth

Rubber infill net bulk density could not be estimated for the field sites since the mass and depth of rubber per unit area of the pitches were unknown and thus total infill depth (sand plus rubber) was used. However, assuming minimal change in sand depth at each location between the compacted (pre-maintenance) and decompacted (post-maintenance) states, the changes in total infill depth could be assumed to approximate changes in rubber infill depth resulting from the decompaction. Furthermore, since all site systems were somewhat similar to the laboratory system (Table 3), some inferences regarding change in relative density may be approximated from the change in total infill depth based on the data presented in Figures 3 and 4.

Total infill depth measurements ranged from 27 to 46 mm across the different field sites and test locations (Table 4). All sites gave a significant increase (*p* < 0.000–0.004) in total infill depth following decompaction which ranged from 3 to 4 mm. This increase is somewhat lower than the 10-mm increase in rubber infill depth recorded between the loosest and densest infill state in the laboratory system, suggesting that the range of compactive states observed on site was somewhat less than the extremes achieved under laboratory conditions.

#### FR

Although all sites displayed a trend of increased FR between the pre- and post-decompaction maintenance, only for site W was this increase significant (*p* = 0.01; Table 4). Furthermore, in all cases the change in FR (average of second and third impacts) was small (between 0.6% and 1.3%). In support of the laboratory data, the field data demonstrated a strong positive relationship between total infill depth and FR (R = 0.51, *p* = 0.015; Figure 5(b)). The lesser strength of this relationship compared to that observed between rubber infill net bulk density and FR in the laboratory is likely due to the data being from four different 3G systems as well as potential confounding influences of contamination, moisture and temperature differences between sites. When looking at the change in FR between the 1st and nth impacts (n = 2→10), the data suggested absolute changes of 6.5%–9.5% across the five locations on the CH site which showed minimal change following decompaction maintenance (Figure 6(b)). Similar trends were reported at the three remaining sites.

The results described above, in combination with the smaller overall range in FRs recorded from a single test position across all four field sites (54%–65%) compared to that for the single laboratory system (50%–67%), again suggest that the field changes in infill state resulting from the decompaction process were somewhat less than the extreme range that can be achieved in the laboratory. Furthermore, the impact energies of the CT and AAA may be too high to afford the sensitivity required to pick up the smaller changes in infill state in the field resulting from decompaction maintenance.

#### VBR

All sites displayed a statistically significant decrease in VBR between the pre- and post-decompaction maintenance (*p* < 0.000–0.004). The magnitude of this decrease ranged from 0.04 m (sites CR and B) to 0.18 m on site W (Table 4). It is unclear as to magnitude of changes in ball bounce that may become meaningful to the users, however. In support of the laboratory data, the field data demonstrated a strong negative relationship between total infill depth and VBR (R = 0.576, *p* = 0.005; Figure 7(b)). The lesser strength of this relationship compared to that observed between rubber infill net bulk density and VBR in the laboratory is likely due to the data being from four different 3G systems as well as potential confounding influences of contamination, moisture and temperature differences between sites.

Once again, the results described above, in combination with the smaller change in VBR in a single test position as a result of the decompaction process recorded across all four field sites (−0.01 to −0.31 m) compared to that for the single laboratory system (−0.41 m), points to the change in infill state resulting from the decompaction process in the field being somewhat less than the extreme range that can be achieved in the laboratory. However, in contrast to FR, the VBR appeared more sensitive to the change in state of the infill resulting from the decompaction maintenance. Based on the laboratory findings, the 0.18-m change in VBR for site W suggests that the working range of infill state for this pitch is at the lower end of rubber infill net bulk density. The smaller changes in VBR for site CR and B suggest that either these pitches are operating in a more compacted state, or that the less intensive maintenance procedure had a measurably lesser decompaction effect. Regardless, it appears that the impact characteristics for VBR are better suited to detecting changes in infill state compared to the FR measurements.

## Discussion

The published literature relating to AGP hardness and the industry views on the role and effects of maintenance collectively suggest that compaction of the rubber infill is expected to occur through mechanical working – caused by the players/users and from trafficking by maintenance plant or other surface loading factors. The compaction leads to hardening of the surface, associated with higher impact forces for player–surface contact (i.e. lower FR from mechanical tests), and increased VBR. If the surface becomes too hard, it may affect the user performance/safety and potentially fail the field accreditation tests (e.g. FIFA^6^) and require some investment to bring it back to a satisfactory standard. The maintenance processes targeted at reversing this hardening effect, by ‘decompacting’ the infill, use bespoke machinery with metal tines to agitate and loosen the infill. The literature to date corroborates that compaction can occur and systems become harder, although few have attempted to measure the infill state or utilise the phenomenon to investigate field behaviour and changes afforded by maintenance.

### Laboratory testing

The laboratory surfaces provided well-controlled and idealised samples for investigating compaction as, unlike the field installed systems, they remained dry and contaminant free and infill net bulk densities could be estimated with confidence.

The laboratory data give a strong relationship between the number of cycles of the roller compactor and the rubber crumb infill depth, and hence net bulk density of the rubber infill. The assumption that the sand is incompressible is a reasonable one as the particles are rigid, round and initial rolling of the sand layer alone led to negligible changes in depth.

The range of net infill bulk densities determined suggested a ‘loose’ state to be defined by values of approximately 0.45–0.50 g cm^−3^, and for a ‘dense’ state density values of 0.65–0.70 g cm^−3^. The loose state in the laboratory was achieved either by hand application with minimal conditioning with the compaction roller or by decompaction by raking after compaction. The lower density value of 0.45 g cm^−3^ is similar to values reported previously^3^ and is similar to the standard bulk density test^18^ used for estimating infill density in a steel mould for quality control. This suggests the decompacted density achieved in the laboratory represents the infill in its loosest state. Particle packing theory would suggest this density value for the loosest state will be affected by particle shape, range of sizes and the intrinsic particle density. In the United Kingdom, the rubber infills used tend to be in the range of 0.5–2.5 mm in size, shredded through an ambient process and comprise recycled truck tyres.

The densest rubber infill state was achieved by multiple compaction cycles (up to 500) with the compaction roller, and/or by 10 repeated impacts with the AAA impact test apparatus. The concept of a relative density range to describe the in situ density state on a scale of 0%–100% is suggested as a useful tool, representing a range of approximately 0.45–0.70 g cm^−3^ from this study. The upper density state has only been reported in similar previous work^3^ in relation to the effect of density state on traction resistance, which reported 0.48–0.65 g cm^−3^ bulk density for similar carpet system, but it is noted the roller compaction took place with the infilled carpet placed on a thick (15–30 mm) shockpad which may account for the lower maximum recorded infill net bulk density.

To achieve a change in relative density from 0% to 40% required around 50 cycles of the compaction roller, whereas to achieve 100% required a further 400 cycles (Figure 3). This compactability behaviour is akin to the well-established behaviour of soil during compaction,^22^ even though the rigidity of the particles is very different. This upper limit of achievable density is controlled by the same issues of particle packing theory as the loose state, in combination with the amount of compactive effort applied, often expressed as compaction energy.^22^

In terms of the laboratory hardness testing, the extremes of density achieved translated into an approximate FR range of 66%–50% (loose to dense). The standard AAA FR test method (average of second and third impacts) gives a slightly narrower range of 62%–57%. As expected, the denser the rubber infill, the harder the surface (lower FR) measured with the AAA (i.e. greater peak deceleration of the falling mass). This shows, however, that during the current standard AAA test method, the infill is being compacted and the initial density state is changing during the three impacts applied (standard test). Table 4 shows this change in more detail, and that the change can be several percentage points of FR for the three impacts. Furthermore, the magnitude of change in FR between the first and third impacts is affected by the initial density state. The consequence is that this standard ‘hardness’ test method will not lead to a standard hardness response in the sample under test. The use of 10 repeated impacts of the AAA on the same test spot is suggested as useful in determining the potential of the infill to be compacted further – hence giving some indication of the initial state relative to the fully compacted state.

The FR measures the impact response of the system upon loading. The VBR, in contrast, measures the recoverable energy on unloading (partly from the energy restitution response of the surface and partly from the pressurised ball’s energy recovered after its maximum deformation). This combined effect can be expressed as the coefficient of restitution and depends on the initial test conditions (primarily ball pressure, drop height/impact velocity). For the VBR test in the laboratory, it was observed that changes in the initial rubber net bulk density had a large effect on VBR height. For the loose infill state, the rebound height was lower, interpreted as greater energy loss (hysteresis) in the infill during loading/unloading due to more plastic straining occurring under the same load compared to when the infill is in a denser state. However, the relationship was non-linear (Figure 7) such that VBR height increased from 0.50 to 0.85 m for density changes of 0.45 g cm^−3^ to approximately 0.55 g cm^−3^ and then only slightly from 0.85 to 0.95 m for infill net bulk densities above approximately 0.55 g cm^−3^ (or >40% relative density). The laboratory data, in combination, provide a useful framework for understanding the density state of the infill and the influence changes in its state can have on the system hardness response to impact testing.

### Field testing

The field systems evaluated in this study comprised long pile sand and rubber infilled systems (3G) without a shockpad. The range of FR hardness values (using the standard method of the averaging the second and third impacts) gave values in the range of 51%–57%, which is at the low end of the accepted FIFA 1* range, suggesting the pitches are relatively hard. In addition, large variability in FR was observed across each; the maximum difference was 9% (at site B) and the minimum 5% (at site W). The average VBR values were in the range of 0.82–0.99 m in a ‘compacted’ state; for each site, the maximum difference after decompaction was 9 cm (at site W) and minimum 2 cm (at site CH). Neither FR nor VBR showed a strong relationship with infill depth, in contrast to the laboratory findings. However, in the laboratory, changes in infill depth were only due to compacted density state, while in the field infill depth may be a function of both infill quantity and density state as well as carpet specification (e.g. fibre size, tuft spacing, texture).

It was anticipated that the decompaction process utilised on the fields would instigate changes in the in situ density state and concomitant changes in play performance measurements of FR and VBR. It was observed from the field data that in general at all sites the decompaction process created an increased depth of infill, suggesting an immediate reduction in the density state of the rubber infill (assuming no changes in the sand layer beneath). These changes in infill depth of 3–4 mm were much smaller than the large depth changes observed in the laboratory of 10 mm, further suggesting that the initial in situ infill density was either at the low end of the relative density range, such that further loosening was only partially achievable, or that the decompaction process was only slightly effective. The play performance data further added to this interpretation but were somewhat less conclusive. In all test locations at each field, the decompaction process led to a slight increase in the FR (i.e. slightly softer surface) and a reduction in the VBR (i.e. softer and less resilient surface). Site CH had a total infill depth close to that utilised in the laboratory sample, albeit with a slightly shorter carpet pile and demonstrated a large change in VBR (average: 10 cm, range: 6–17 cm); in contrast, the FR changed very little. In contrast, site B with total infill depth similar to CH (and the laboratory sample) experienced only a very small change in VBR (average: 4 cm) and again little change in FR. Site W had the most vigorous decompaction and notably the highest recorded change in VBR (average: 18 cm, range: 6–31 cm). The total infill depth at site W was, however, the smallest, thus a relatively hard system response would be expected (there is no shockpad beneath) and the absolute values for VBR and FR support this, regardless of infill density state. The large change in VBR at site W does suggest an appreciable change in the infill density based on the laboratory data, although again there was little observed change in FR.

It is suggested from these data that the VBR is sensitive to the small changes in rubber infill depth caused by a change in density state, and the FR from CT/AAA is more affected by the whole system of rubber/sand/substrate. Furthermore, it is also clear from this study that the AAA/CT impacts promote densification of the rubber infill under subsequent impacts such that the standard reporting of the average of the second and third impacts is potentially measuring a higher infill density-related response than the true initial state (i.e. that experienced by the players). Furthermore, it is useful to note that by expressing the compaction energy of each test as the potential energy per unit area^23^ gives value of 2.6 kJ m^−2^ for the AAA, 4.5 kJ m^−2^ for the CT and 0.9 kJ m^−2^ for the VBR, supporting the argument that the VBR test will cause a smaller change in density state during testing on compactable materials.

It was apparent from the laboratory testing that the largest change in in situ density, and changes in VBR and FR, occurred in the lower relative infill density range of 0%–40% and this was achieved by relatively light compactive effort (i.e. few cycles of the studded roller). The field data, in combination, suggest that it is unlikely that the very loose state is achieved after decompaction, and further that the initial field state is not at the very densest state achieved in the laboratory. The direct comparison of the field data to the laboratory data is, however, confounded by the variation in infill depth observed and the extraneous factors that can influence the field test hardness data, such as the ratio of sand to rubber, contaminant materials in the infill (changing the packing and density behaviour), moisture state and temperature differences during the testing. The effects of these variables are currently not very well defined or understood. However, it has been observed that contamination (often fines such as silt, broken fibres and decaying organic matter) can block the void spaces leading to poorer infiltration and a ‘harder’ surface^14^ particularly during dry periods, but may also retain more moisture during wet periods such that the surface reacts more softly. In general, temperature increases would be expected to soften the rubber infill and reduce ball bounce and FR, though little work permits an estimate of the size of this effect. For a well-drained pitch, moisture has been observed to have little effect on impact testing for hardness such as FR,^3^ but some effect on dissipating ball impact energy (e.g. in hockey) especially if very wet. Clearly, further work exploring the VBR and FR relationships with infill state is warranted, including further correlations for VBR and density for a range of infill depths, and what adjustments are required for variation in temperature and water content. The role of the carpet fibres in the behaviour observed is also unknown and while it may be assumed that the fibre role in resistance to vertical load is minimal, they have a role in ‘containing’ the infill such that a high density/frequency of fibres may be expected to provide greater resistance to infill movement. In compression, it may be considered that the fibres will act to resist horizontal strain in the infill layer such that vertical deformation is reduced – in effect, the fibres contribute to a higher Poisson’s ratio in terms of elastic theory.

The nature of infill compaction in a newly installed field is an important factor when considering the findings of the field data. During the in-service life of these sport surface systems, the mechanical compaction is afforded by the players/users but there is also the potential for agitation through movements such as stud shearing interactions and kicking. As a consequence, it is suggested that the field state of the infill may be expected to exhibit a smaller density range than that created in the laboratory. Regular maintenance may potentially reduce this range further.

### Role of maintenance and implications

The field results from these regularly maintained AGPs suggest broadly that the in situ infill density state is being maintained at a looser rather than denser state. The standard decompaction process (i.e. two passes of the plant plus surface brushing to even out infill depths) carried out regularly maintains a suitable state of infill and helps regulate, in general, the play performance. In practice, this process is usually coincident with cleaning and brushing to ensure the effect of these interventions on the site availability and cost is kept to a minimum. It is suggested that a second more invasive and intensive process of decompaction should be considered for reactive maintenance on neglected fields. The more invasive process should make several passes over the surface, ideally agitating the full depth of the rubber infill.

The field should be tested to quantitatively confirm changes in play performance and help determine the appropriate intervention process required. It is suggested that in these AGP assessments, 10 impacts from the AAA/CT should be carried out, analysing the change in FR from the 1st to the 10th impact to assess the ‘compactability’ of the infill. The smaller the difference between the 1st and 10th impact, the greater the need for a decompaction process. The VBR test is also considered appropriate, and it would appear more sensitive to changes in the infill state. In combination, these two tests can form a useful diagnostic tool kit to support decisions regarding the maintenance regime. In addition, frequent measurements of infill depth would potentially establish compaction occurring, although it is observed that large quantities (a few millimetres over the large surface area) of rubber can be removed by the users and heavy rain, for example, requiring top ups. However, clearly the specification of the AGP system, the infill depth and its initial state will affect the potential post-maintenance recovered performance, as demonstrated in this study. In general, it is suggested that an increased mass/depth of rubber infill will exhibit greater changes in state if they are initially in a denser state than systems with less infill. The ideal scenario would thus be for relatively frequent monitoring of the AGP soon after installation to assess the general degradation in performance over the in-service life of the asset, perhaps on a 6-monthly basis.

## Conclusion

Little existing research has focussed on investigating the state of the infill and its contribution to hardness, with regard to explaining the mechanisms and attempting to measure the changes that can occur in the field. The effectiveness of maintenance practices in reducing this form of performance loss or prolonging the life of the installed system is also under researched. However, it is clear from past research that the density of the infill changes and can be utilised to help explain changes in play performance, particularly FR and VBR. Determining infill net bulk density should be considered for future hardness-related testing as an important variable affecting play performance, and for controlling carefully when identifying the effects of other variables on play performance, particularly in the laboratory assessment of system behaviour.

Field measurements during the maintenance decompaction process have shown the value of recording the changes in VBR and FR, including 10 impacts of the AAA/CT to determine the full hardening behaviour of the surface. The in situ state of the infill in the field was evaluated through back analysis of the changes in total infill depth. However, the unknown depth of the separate sand and rubber layers individually remains a confounding variable for benchmarking the amount of change observed in the field.

Future decompaction maintenance may benefit from more intensive agitation of the infill, dependent on the initial state. Further work on assessing the effects of 10 impacts of AAA/FR and VBR measurements across a range of rubber infill depths and system designs will allow for the determination of benchmark values for what is considered a ‘compacted’ or ‘loose’ state in field measurements. This will better allow the state of the infill to be determined routinely through regular site inspections and to determine the intensity of the decompaction process required.
